# Non-Sterilized Fermentation of 2,3-Butanediol with Seawater by Metabolic Engineered Fast-Growing *Vibrio natriegens*

**DOI:** 10.3389/fbioe.2022.955097

**Published:** 2022-07-12

**Authors:** Wensi Meng, Yongjia Zhang, Liting Ma, Chuanjuan Lü, Ping Xu, Cuiqing Ma, Chao Gao

**Affiliations:** ^1^ State Key Laboratory of Microbial Technology, Shandong University, Qingdao, China; ^2^ State Key Laboratory of Microbial Metabolism, Joint International Research Laboratory of Metabolic and Developmental Sciences, School of Life Sciences and Biotechnology, Shanghai Jiao Tong University, Shanghai, China

**Keywords:** water resource, freshwater, energy efficient, salt tolerance, microbial chassis, platform compound

## Abstract

Sustainable and environment-friendly microbial fermentation processes have been developed to produce numerous chemicals. However, the high energy input required for sterilization and substantial fresh water consumption restrict the economic feasibility of traditional fermentation processes. To address these problems, *Vibrio natriegens*, a promising microbial chassis with low nutritional requirements, high salt tolerance and rapid growth rate can be selected as the host for chemical production. In this study, *V. natriegens* was metabolic engineered to produce 2,3-butanediol (2,3-BD), an important platform chemical, through non-sterilized fermentation with seawater-based minimal medium after expressing a 2,3-BD synthesis cluster and deleting two byproduct encoding genes. Under optimized fermentative conditions, 41.27 g/L 2,3-BD was produced with a productivity of 3.44 g/L/h and a yield of 0.39 g/g glucose by recombinant strain *V. natriegens*Δ*frdA*Δ*ldhA*-pETRABC. This study confirmed the feasibility of non-sterilized fermentation using seawater to replace freshwater and other valuable chemicals may also be produced through metabolic engineering of the emerging synthetic biology chassis *V. natriegens*.

## Introduction

Traditional petrol-based industry has contributed most of the chemicals in our daily lives (Levi and Cullen, 2018). Microbial fermentation producing chemicals from renewable resource is an environmentally friendly and sustainable way to replace the petrol-based industry (Dessie et al., 2020; Ramamurthy et al., 2021). However, typical microbial fermentation often faces the problem of easy contamination and thus requires complicated sterilization and high energy input (Li et al., 2014; Yu et al., 2019; Guo et al., 2020a). In addition, it also consumes a large amount of freshwater for preparing the culture medium (Yue et al., 2014). Identifying a suitable microbial strain excluding expensive sterilization and fresh water consumption is highly desirable for development of an energy efficient and sustainable biotechnology.

*Vibrio natriegens* is a nonpathogenic marine bacterium with a doubling time of 9.8 min (Eagon, 1962; Hoff et al., 2020). It has characteristics of low nutritional requirements, broad substrate spectrum, and robust growth under high salt condition (Hoffart et al., 2017; Hoff et al., 2020). Recently, the development of gene manipulation methods and tools of *V. natriegens* makes it possible to use this strain as a candidate host for microbial cell factory construction (Weinstock et al., 2016; Dalia et al., 2017; Hoffart et al., 2017; Tschirhart et al., 2019). *V. natriegens* has been metabolic engineered to produce a series of compounds such as alanine, beta-carotene, violacein, melanin, and 1,3-propanediol (Hoffart et al., 2017; Ellis et al., 2019; Wang et al., 2020; Zhang et al., 2021). Seawater is a widely available water source. *V. natriegens* grows quickly in marine and exhibits high salt tolerance. In this work, we identified the feasibility of non-sterilized fermentation by *V. natriegens* using seawater as an alternative of freshwater.

**FIGURE 1:**
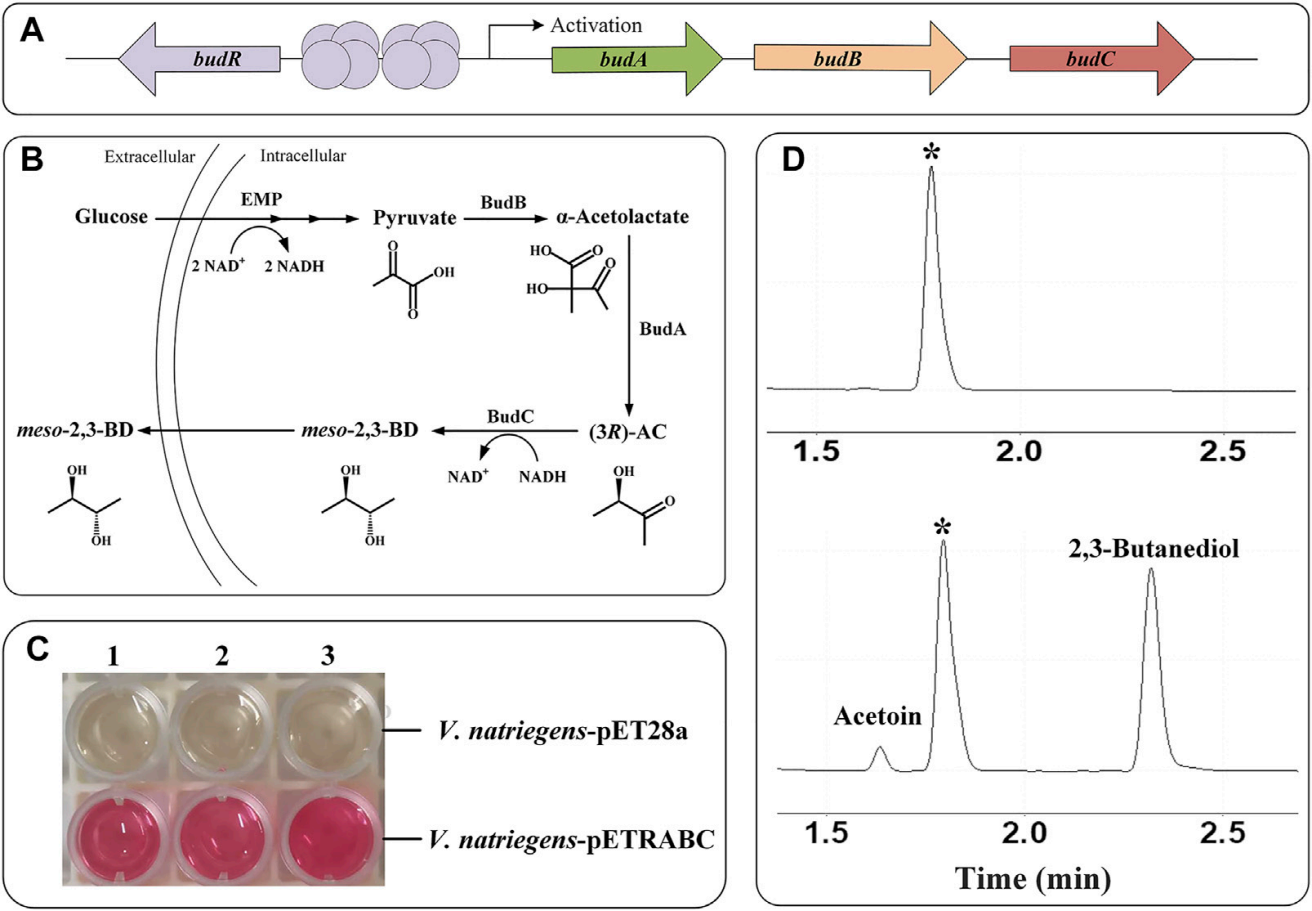
Verification of the ability of the recombinant *V. natriegens* expressing *budRABC* to metabolize glucose into 2,3-BD **(A)** Structure of the gene cluster *budRABC* in *E. cloacae* SDM responsible for 2,3-BD production **(B)** Metabolic process of 2,3-BD production from glucose in *V. natriegens* with expressed 2,3-BD synthesis pathway **(C)** VP test result of *V. natriegens*-pET28a and *V. natriegens*-pETRABC **(D)** Chromatograph profile of 2,3-BD produced from glucose using *V. natriegens*-pET28a (up) and *V. natriegens*-pETRABC (down). *Isoamylol was used as the internal standard.

2,3-Butanediol (2,3-BD) is an important platform compound with diversified applications in many industrial fields (Ge et al., 2016; Meng et al., 2020). During 2,3-BD fermentation in *Enterobacter cloacae* subsp. *dissolvens* SDM, 2,3-BD is produced from pyruvate via α-acetolactate synthase (BudB, AFM58914), α-acetolactate decarboxylase (BudA, AFM58913), and *meso*-2,3-BD dehydrogenase (BudC, AFM58915) (Meng et al., 2021). In a previous study, introducing the recombinant plasmid pETRABC expressing BudA, BudB, BudC, and their regulatory protein BudR (AFM58912) (Figure 1A) into *E. coli* realized heterologous synthesis of 2,3-BD from glucose (Xu et al., 2014). In this work, the plasmid pETRABC was firstly transferred into *V. natriegens* type strain ATCC 14048 to obtain recombinant strain *V. natriegens*-pETRABC. Then, the feasibility of non-sterile fermentation using seawater with this strain was identified. After fermentation condition optimization and byproduct pathways deletion, the 2,3-BD production performance of recombinant *V. natriegens* was further improved.

## Materials and Methods

### Enzymes and Chemicals

FastPfu DNA polymerase and T4 DNA ligase were purchased from TransGen Biotech (Beijing, China) and Thermo Scientific (Lithuania), respectively. Restriction enzymes were purchased from TaKaRa Bio Inc (Dalian, China). Primers for polymerase chain reaction (PCR) were synthesized by Tsingke Biology Co., Ltd (Qingdao, China). Racemic acetoin (AC) was purchased from Apple Flavor & Fragrance Group (Shanghai, China) while 2,3-BD was purchased from ACROS (The Kingdom of Belgium). Sodium acetate was purchased from Sinopharm Chemical Reagent Co., Ltd (Shanghai, China). Seawater was taken from the Huanghai Sea near the east gate of Shandong University with a salinity about 30‰ (Aoshan Bay, Qingdao, China). All other chemicals were of analytical grade and commercially available.

### Bacterial Strains, Plasmids and Culture Medium

The strains and plasmids used in this study are listed in Supplementary Table S1. All engineered strains used in this work are based on *V. natriegens* ATCC 14048. The plasmid pETRABC was used for 2,3-BD synthesis while the plasmid pET28a was used for blank control. *E. coli* DH5α was used to hold these two plasmids. The plasmid pKR6K_Cm_ was used for gene knockout and *E. coli* S17-1 was used to hold the plasmid pKR6K_Cm_ (Xin et al., 2017) as well as for conjugation with *V. natriegens*.

Luria-Bertani (LB) medium was used for the cultivation of *E. coli* S17-1 while LB3 medium (5 g/L yeast extract; 10 g/L tryptone; 30 g/L NaCl) was used for the cultivation of *V. natriegens*. The MR-VP medium supplemented with 30 g/L NaCl (5 g/L glucose; 7 g/L peptone; 5 g/L K_2_HPO_4_; 30 g/L NaCl) was used for Voges-Proskauer (VP) test and subsequent gas chromatography (GC) detection. The VN minimal medium (5 g/L (NH_4_)_2_SO_4_; 15 g/L NaCl; 1 g/L KH_2_PO_4_; 1 g/L K_2_HPO_4_; 0.25 g/L MgSO_4_; 0.01 g/L CaCl_2_; 16.4 mg/L FeSO_4_·7H_2_O; 10 mg/L MnSO_4_·H_2_O; 0.3 mg/L CuSO_4_·5H_2_O; 1 mg/L ZnSO_4_·7H_2_O; 0.02 mg/L NiCl_2_·6H_2_O) prepared using distilled water or seawater supplemented with glucose was used in shake flasks experiments for fermentation condition optimization of *V. natriegens* (NaCl is unnecessary when preparing with seawater). The selection medium for single exchange strains of *V. natriegens* was VN minimal medium supplemented with 10 g/L glucose and 40 μg/mL chloramphenicol. The selection medium for double exchange strains of *V. natriegens* was solid LB3 medium supplemented with 15% sucrose.

### Plasmid Transformation of *V. natriegens*

Procedures for competent cell preparation and electrotransformation were according to the method of Weinstock and colleagues (Weinstock et al., 2016) with slight modification. The competent cells of *V. natriegens* were grown in brain–heart infusion medium supplemented with KCl and MgCl_2_ (BHI + v2) at 30°C to OD_600nm_ of 0.5 and immediately moved onto ice for 15 min. Then, the cells were washed three times with cold electroporation buffer (680 mM sucrose, 7 mM K_2_HPO_4_, pH 7.0) and resuspended to a final OD_600nm_ of 16. A Bio-Rad Micro-Pulser and a 1 mm electroporation cuvette were used in electrotransformation. The instrument was set to 900 V, 25 μF, 200 Ω. The transformants of *V. natriegens* were then screened from the BHI + v2 salt solid plate supplemented with 50 μg/mL kanamycin at 37°C.

### Knockout of the Genes in *V. natriegens*

The primers used for gene knockout in *V. natriegens* ATCC 14048 are listed in Supplementary Table S2. DNA manipulations such as vector isolation and restriction enzyme digestion were carried out following standard protocols (Sambrook and Russell, 2001). The method for gene knock-out through allele exchange using the suicide plasmid pKR6K_Cm_ was conducted as described in the previous report (Meng et al., 2020). Briefly, the upstream and downstream homologous arms of *frdA* were amplified from *V. natriegens* ATCC 14048 and then ligated through PCR to get Δ*frdA* fragment using primer pairs PΔ*frdA*.f1 (EcoRI)/PΔ*frdA*.r2 (overlap) and PΔ*frdA*.f3 (overlap)/PΔ*frdA*.r4 (BamHI), respectively. The gel-purified Δ*frdA* fragment was ligated to the pKR6K_Cm_ digested with EcoRI and BamHI. The resulting plasmid pKDΔ*frdA* was introduced into *E. coli* S17-1. Then, a three-step deletion procedure was applied to select the Δ*frdA* mutant after conjugating the pKDΔ*frdA* in *V. natriegens* ATCC 14048 as described previously (Xin et al., 2017). The *ldhA* mutant of strain *V. natriegens* were generated by using the same procedure.

### Batch and Fed-Batch Fermentations

Batch fermentations were conducted in a 1-L bioreactor (Multifors 2, Infors AG, Switzerland) with 0.8 L of medium. The seed culture was inoculated (10%, v/v) into the seawater-based VN minimal medium supplemented with 50 g/L glucose. The cultivation was carried out at 37°C, airflow at 1.0 vvm, stirring at 400 rpm. The initial pH was set to 7.5 and maintained by automatic addition of 5 M NaOH. Fed-batch fermentation was carried out under the same cultivation condition in a 7.5-L fermenter (BioFlo 310, NBS, United States) containing 5 L of medium. Solid glucose was added when residual glucose concentration was reduced to about 10 g/L.

### Analytical Methods

The optical density (OD) was measured at 600 nm using a spectrophotometer V5100H and the concentration of glucose was determined by a bioanalyzer SBA-40D after diluting to an appropriate concentration. The concentrations of by-products including succinate, lactate, formate, acetate, and ethanol were determined by using Agilent 1,100 equipped with an Aminex HPX-87H column (300 mm× 7.8 mm; Bio-Rad, United States) and a refractive index detector (Meng et al., 2020). The concentrations of AC and 2,3-BD were measured by GC (Shimadzu, GC 2014c) using a capillary GC column as described previously (Ge et al., 2016).

## Results and Discussion

### Expression of 2,3-BD Synthetic Gene Cluster in *V. natriegens*

*V. natriegens*-pETRABC was constructed through transferring the plasmid pETRABC into *V. natriegens* type strain ATCC 14048 and cultured in MR-VP rich medium. Theoretically, glucose will be first metabolized through EMP pathway in *V. natriegens* to produce the center metabolite pyruvate (Hoff et al., 2020). Then, overexpressed BudA, BudB and BudC can catalyze pyruvate to form 2,3-BD via intermediates α-acetolactate and AC (Figure 1B). As shown in Figures 1C,D, the results of Voges-Proskauer (VP) test and gas chromatography indicated that *V. natriegens*-pETRABC harboring 2,3-BD synthesis pathway has the ability to metabolize glucose in MR-VP medium to produce 2,3-BD.

### Non-Sterile 2,3-BD Fermentation by *V. natriegens*-pETRABC Using Seawater

*V. natriegens* can also rapidly metabolize glucose in minimal medium containing inorganic salt ions (Hoffart et al., 2017). Thus, we explored the feasibility of using VN minimal medium (Hoffart et al., 2017) to cultivate *V. natriegens*-pETRABC for 2,3-BD production. In order to further reduce the fermentation cost and save fresh water, the possibility of producing 2,3-BD through non-sterilized fermentation using seawater was analyzed. Since the seawater contains about 3.0% NaCl, the addition of external NaCl in VN medium is unnecessary. Four different conditions were set for bacterial culture: distilled water-based VN minimal medium with sterilization (Medium 1) or without sterilization (Medium 2); seawater-based VN minimal medium with sterilization (Medium 3) or without sterilization (Medium 4). The concentration of glucose was set to 40 g/L and the cell growth, sugar consumption, concentration and yield of 2,3-BD of *V. natriegens*-pETRABC were detected for comparing the performance of the recombinant strain in different media.

**FIGURE 2:**
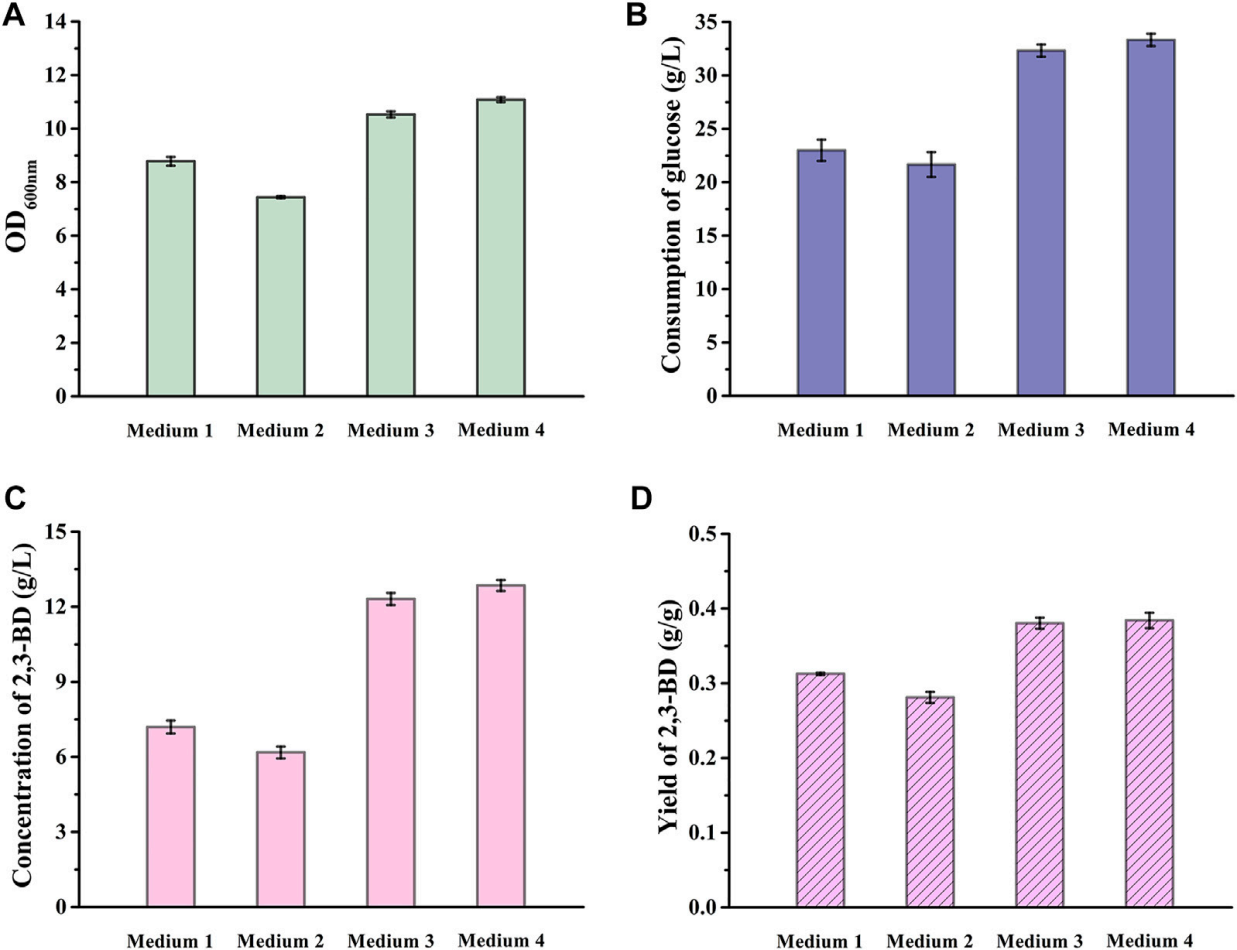
Performance of *V. natriegens*-pETRABC in different media. Biomass **(A)**, consumption of glucose **(B)**, concentration **(C)** and yield **(D)** of 2,3-BD using glucose as the carbon source by *V. natriegens*-pETRABC were assayed. The experiments were conducted in 300-mL flasks containing 50 mL of different media supplemented with 40 g/L glucose at pH 7.0. Data shown are mean ± s.d. (*n* = 3 independent experiments). Medium 1: distilled water prepared VN minimal medium with sterilization; Medium 2: distilled water prepared VN minimal medium without sterilization; Medium 3: seawater prepared VN minimal medium with sterilization; Medium 4: seawater prepared VN minimal medium without sterilization.

As shown in Figure 2, the cell growth, glucose consumption and 2,3-BD accumulation of *V. natriegens*-pETRABC in the seawater-based VN minimal medium were better than those in distilled water-based VN minimal medium, indicating that the high salt and other ingredients in seawater can support the metabolism of *V. natriegens*-pETRABC. *V. natriegens*-pETRABC in non-sterilized seawater medium exhibited almost the same performance as the sterilized one, proving that the high salinity of seawater can inhibit the growth of bacterial contamination and seawater is suitable for fermentation. After 12 h cultivation of *V. natriegens*-pETRABC in Mediun 4, the concentration of 2,3-BD reached 12.85 g/L with a yield of 0.38 g/g glucose. Thus, Medium 4 was selected for further study.

### Optimization of Fermentation Conditions of *V. natriegens*-pETRABC

**FIGURE 3:**
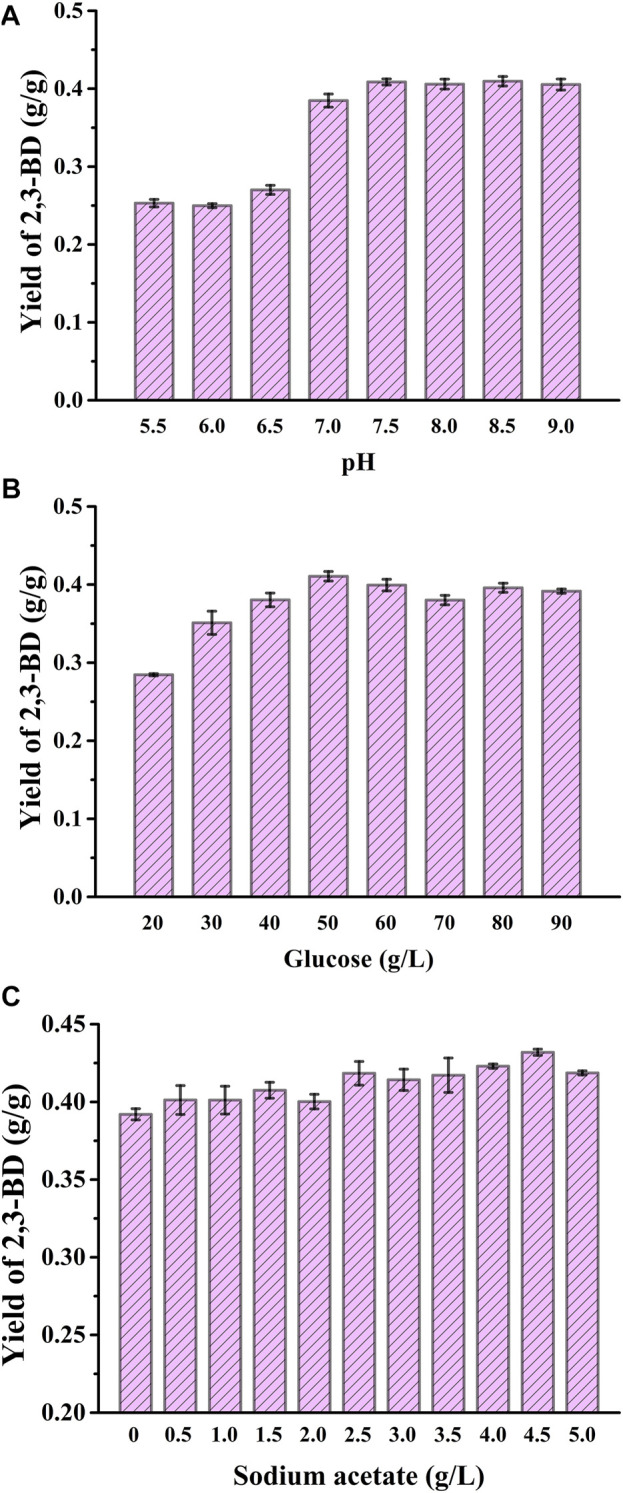
Optimization of pH, initial glucose concentration, and addition of sodium acetate for 2,3-BD production by *V. natriegens*-pETRABC **(A)** Yield of 2,3-BD in media with different pHs **(B)** Yield of 2,3-BD in media with different glucose concentrations **(C)** Yield of 2,3-BD in media with different sodium acetate concentrations. The experiments were conducted in 300-mL flasks containing 50 mL of medium. Data shown are mean ± s.d. (*n* = 3 independent experiments).

In order to further increase 2,3-BD production, the pH of the fermentation system was optimized. The concentration of glucose used was 40 g/L, and the pH was set from 5.5 to 9.0. The cell growth, glucose consumption, the production and yield of 2,3-BD under different pHs were assayed (Figure 3A and Supplementary Figures S1A–C) and 7.5 was selected as the optimal pH. Carbon source concentration was also optimized. Glucose concentration was set from 20 g/L to 90 g/L. *V. natriegens*-pETRABC exhibited good tolerance to even 90 g/L glucose. The highest concentration and yield of 2,3-BD were obtained when the glucose concentration was 50 g/L (Figure 3B and Supplementary Figures S1D–F). Recent studies have shown that adding exogenous sodium acetate at the beginning of cultivation can enhance the transcription level of the 2,3-BD synthetic gene cluster in *E. cloacae* SDM (Meng et al., 2021), thus may have a positive effect on 2,3-BD accumulation of *V. natriegens*-pETRABC. However, acetate at a too high concentration will inhibit the growth of the strain (Thomas et al., 2014). The concentration of sodium acetate was therefore optimized by setting the concentration ranging from 0 g/L to 5 g/L. The highest biomass was obtained at the sodium acetate concentration of 2.5 g/L while the highest 2,3-BD concentration was acquired at the sodium acetate concentration of 4.5 g/L (Figure 3C and Supplementary Figures S1G–I). Thus, the optimal fermentation conditions for *V. natriegens*-pETRABC were using seawater-based VN minimal medium without sterilization, pH of 7.5, initial glucose concentration of 50 g/L, and sodium acetate concentration of 4.5 g/L.

### Deletion Byproduct Pathways to Increase 2,3-BD Production of Recombinant *V. natriegens*

**FIGURE 4:**
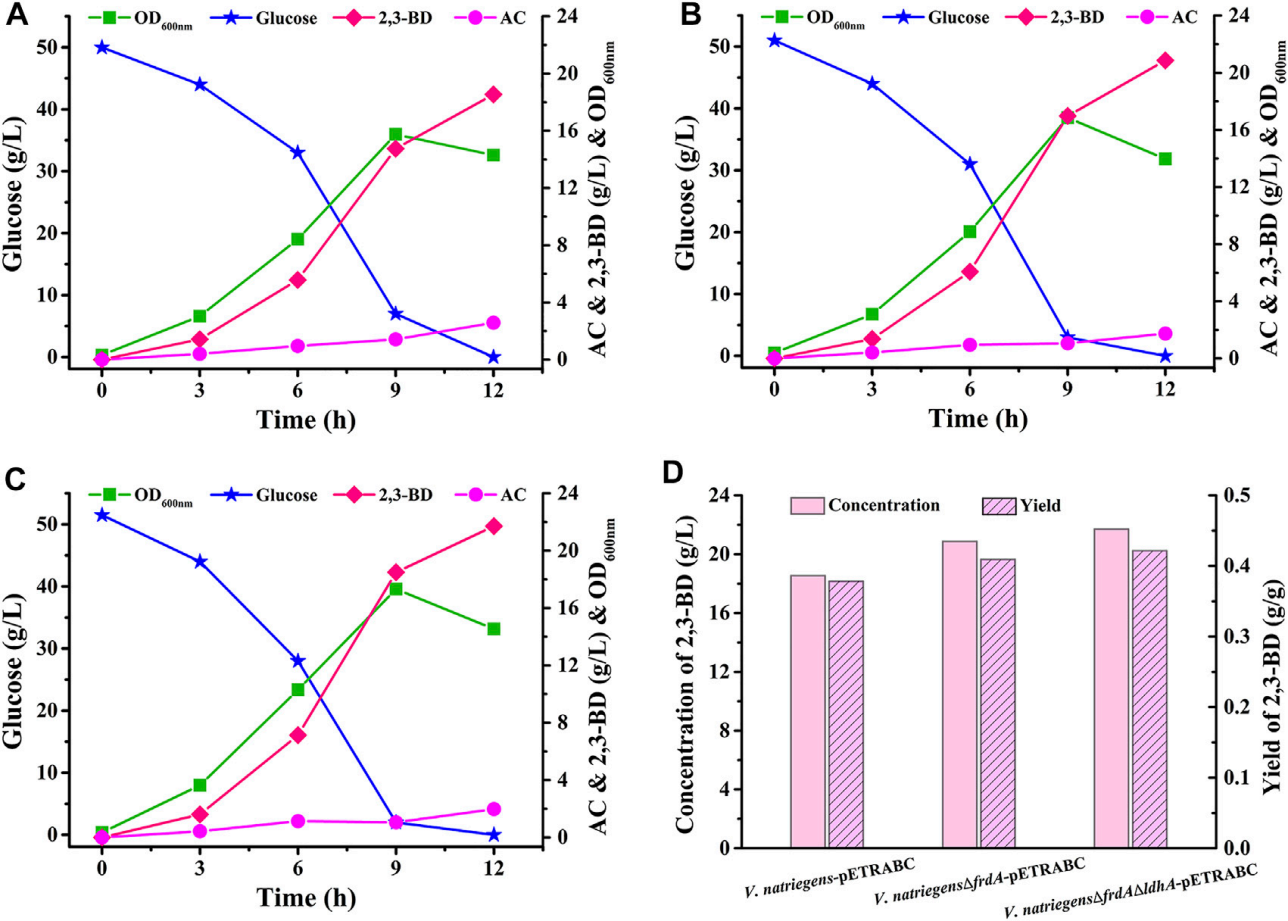
Non-sterilized batch fermentation of *V. natriegens*-pETRABC **(A)**, *V. natriegens*Δ*frdA*-pETRABC **(B)**, and *V. natriegens*Δ*frdA*Δ*ldhA*-pETRABC **(C)** in 1-L fermenter **(D)** Concentration and yield of 2,3-BD produced by *V. natriegens*-pETRABC, *V. natriegens*Δ*frdA*-pETRABC, and *V. natriegens*Δ*frdA*Δ*ldhA*-pETRABC. The experiments were conducted in a 1-L fermenter containing 0.8 L of medium with an initial glucose concentration of 50 g/L.

The 2,3-BD production performance of *V. natriegens*-pETRABC was analyzed in 1-L fermenter (Figure 4A). *V. natriegens*-pETRABC could consume about 50 g/L glucose in 12 h and accumulate 18.54 g/L 2,3-BD. The yield and productivity of 2,3-BD were 0.38 g/g glucose and 1.55 g/L/h, respectively. In addition to the target product 2,3-BD, byproducts including succinate (5.23 g/L), lactate (0.67 g/L), formate (1.36 g/L), and ethanol (2.85 g/L) were also detected (Supplementary Figure S2A). In order to further improve the production performance of *V. natriegens*-pETRABC, we tried to knockout the succinate encoding gene *frdA* and lactate encoding gene *ldhA via* allele exchange using the suicide plasmid pKR6K_Cm_ (Supplementary Tables S1, S2) (Xin et al., 2017). As shown in Figure 4B, after deletion of *frdA*, the 2,3-BD accumulation of *V. natriegens*Δ*frdA*-pETRABC was significantly increased to 20.87 g/L while the concentration of succinate was reduced to only 0.17 g/L (Supplementary Figure S2B). A further deletion of *ldhA* enabled the strain *V. natriegens*Δ*frdA*Δ*ldhA*-pETRABC accumulated 21.71 g/L 2,3-BD with a yield of 0.42 g/g glucose (Figures 4C,D). Meanwhile, succinate and lactate were almost undetectable (Supplementary Figure S2C).

### Fed-Batch Fermentation of *V. natriegens*Δ*frdA*Δ*ldhA*-pETRABC

**FIGURE 5:**
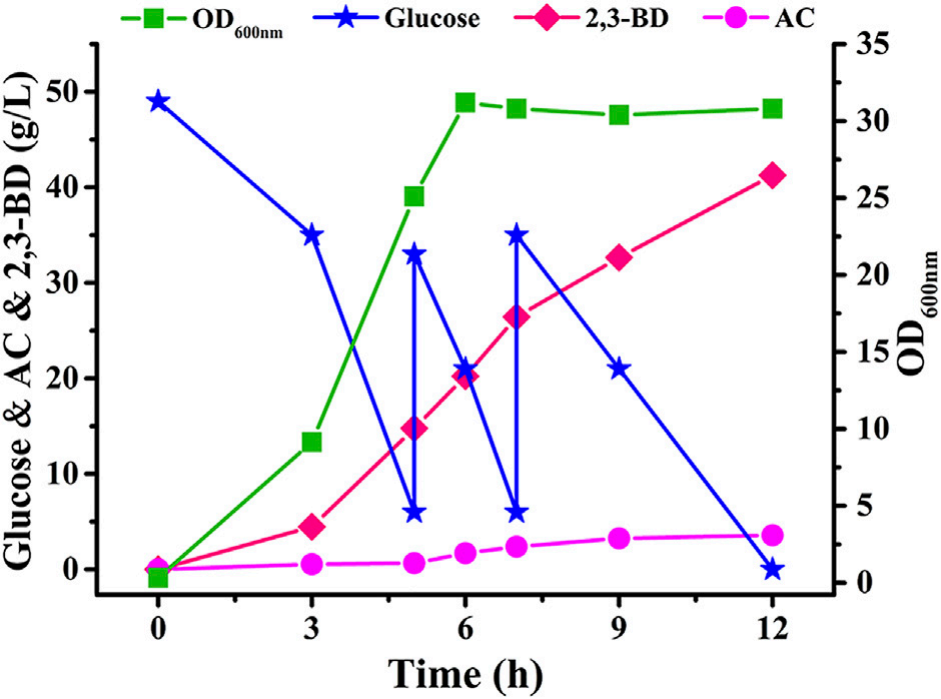
Time course of non-sterilized fed-batch fermentation using *V. natriegens*Δ*frdA*Δ*ldhA*-pETRABC in 7.5-L fermenter. Biomass, consumption of glucose, concentration of AC and 2,3-BD were assayed. The experiments were conducted in a 7.5-L fermenter containing 5 L of medium with an initial glucose concentration of 50 g/L.

Fed-batch fermentation in a 7.5-L fermenter with non-sterilized seawater medium by strain *V. natriegens*Δ*frdA*Δ*ldhA*-pETRABC was also carried out. After 12 h of fermentation, 41.27 g/L 2,3-BD was obtained from 105 g/L glucose (Figure 5). The productivity and yield of 2,3-BD were 3.44 g/L/h and 0.39 g/g, respectively. The major by-products in final fermentation broth were ethanol, formate, and AC, which were found at concentrations of 6.45 g/L, 4.06 g/L, and 3.57 g/L, respectively. A small amount of lactate (1.72 g/L) and succinate (0.45 g/L) were also detected (Supplementary Figure S3).

**FIGURE 6:**
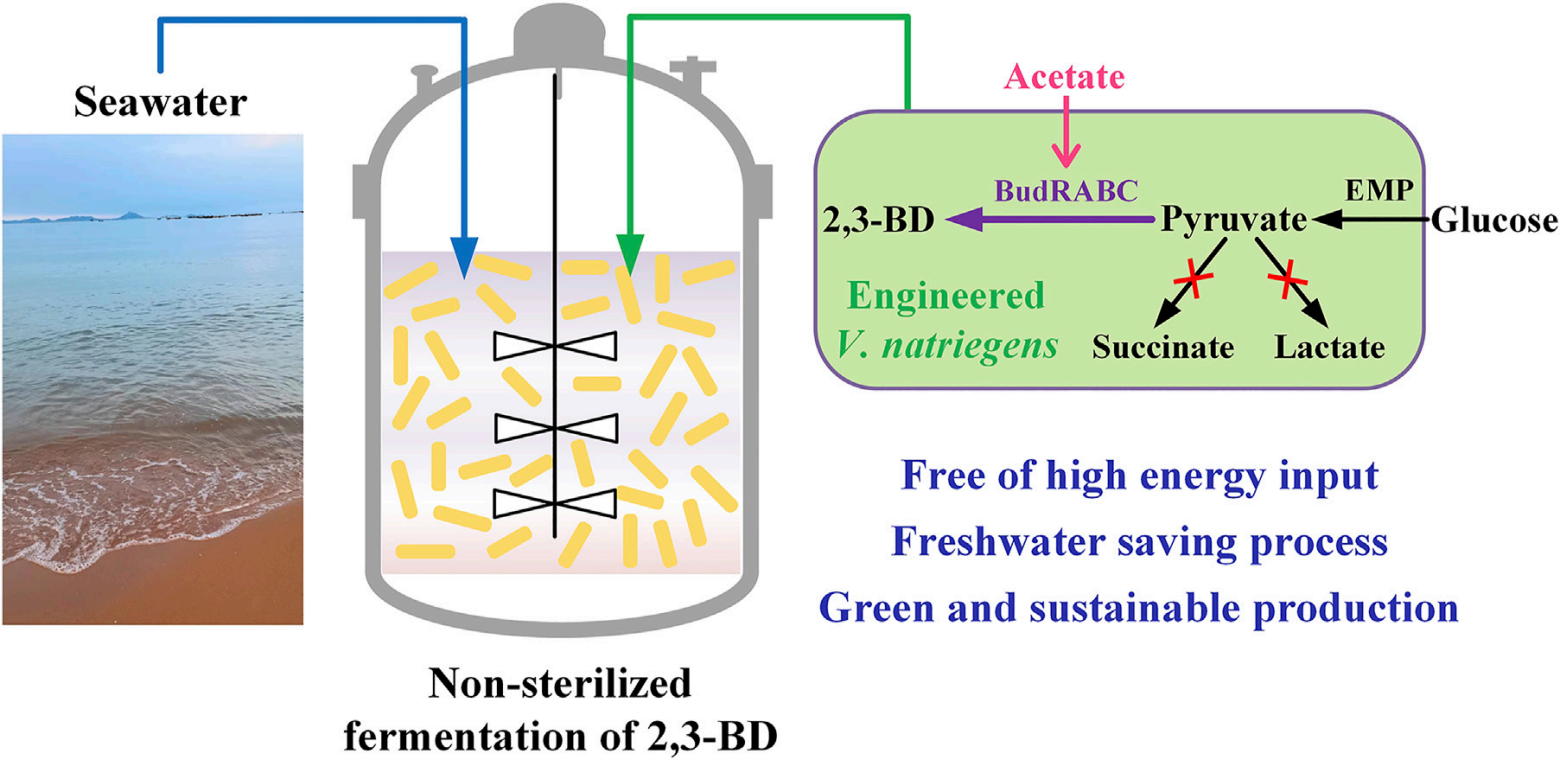
Metabolic engineering of *V. natriegens* for sustainable production of platform chemical 2,3-butanediol through non-sterilized fermentation with seawater.

*V. natriegens* is considered to be a potential chassis for industrial biotechnology due to its advantages of fast growth and carbohydrate metabolism (Peng et al., 2020; Thoma and Blombach, 2021). In this study, we explored the possibility to engineer *V. natriegens* for heterologous production of 2,3-BD from glucose based on seawater in non-sterilized fermentation condition (Figure 6). Strategies including medium optimization and by-products pathways deletion were beneficial in improving the production and yield of 2,3-BD. The biomass of *V. natriegens* showed a significant decrease in the later fermentation stage, indicating the low stability of the strain in long-term cultivation. Conley et al. found that extracellular electron transfer (EET) enhanced survival of *V. natriegens* under fermentative conditions and identified the mechanism of EET in *V. natriegens* (Conley et al., 2020). Addition of extracellular acceptor like Fe(III) citrate or overexpression of CymA, PdsA, and MtrCAB necessary for EET may be beneficial to EET and enhance the fermentative performance of *V. natriegens*.

Erian et al. also attempted to produce 2,3-BD by engineered *V. natriegens*. When cultured in medium without yeast extract, the recombinant strain could accumulate 27.4 g/L diol (2,3-BD plus AC) but AC accounts for about 10 g/L (Erian et al., 2020). In this work, the engineered *V. natriegens*Δ*frdA*Δ*ldhA*-pETRABC was confirmed to have the ability to efficiently transform glucose into 2,3-BD with relatively higher titer (41.27 g/L), high productivity (3.44 g/L/h) and high glucose consumption rate (8.75 g/L/h) in seawater-based VN minimal medium (Figure 5). AC only accumulated at a concentration of 3.57 g/L. *Klebsiella pneumoniae* OU7 can metabolize urea and phosphite as its primary sources of nitrogen and phosphorus. Guo et al. achieved non-sterilized fermentation of 2,3-BD using recombinant *K. pneumoniae* through addition of these unconventional chemicals to provide selective pressure (Guo et al., 2020b). *V. natriegens* is a biosafety bacteria (Risk Group 1) with broad substrate spectrum and excellent production characteristics. Non-sterilized fermentation of 2,3-BD with seawater by *V. natriegens* may help to both reduce energy costs and conserve fresh water resource. Besides 2,3-BD introduced in this work, various chemicals may also be produced by non-sterilized fermentation using seawater through metabolic engineering of *V. natriegens*.

## Conclusion

In this study, *V. natriegens* ATCC 14048 was successfully engineered for 2,3-BD production from glucose by overexpressing the 2,3-BD synthesis gene cluster from *E. cloacae* SDM. The knocking out of two genes *frdA* and *ldhA* decreased the production of byproducts succinate and lactate. The recombinant strain was able to grow in seawater-based VN minimal medium and efficiently produce 2,3-BD through non-sterilized fermentation with a titer of 41.27 g/L and a productivity of 3.44 g/L/h. The process developed in this study is not only an alternative for economic production of 2,3-BD, but also a successful example for production of other chemicals through non-sterilized fermentation with seawater.

## Data Availability

The original contributions presented in the study are included in the article/Supplementary Material, further inquiries can be directed to the corresponding author.

## References

[B1] Conley B. E. Weinstock M. T. Bond D. R. Gralnick J. A. (2020). A Hybrid Extracellular Electron Transfer Pathway Enhances the Survival of *Vibrio natriegens* . Appl. Environ. Microbiol. 86, e01253–20. 10.1128/AEM.01253-20 32737131 PMC7499025

[B2] Dalia T. N. Hayes C. A. Stolyar S. Marx C. J. McKinlay J. B. Dalia A. B. (2017). Multiplex Genome Editing by Natural Transformation (MuGENT) for Synthetic Biology in *Vibrio natriegens* . ACS Synth. Biol. 6, 1650–1655. 10.1021/acssynbio.7b00116 28571309 PMC6519440

[B3] Dessie W. Luo X. Wang M. Feng L. Liao Y. Wang Z. (2020). Current Advances on Waste Biomass Transformation into Value-Added Products. Appl. Microbiol. Biotechnol. 104, 4757–4770. 10.1007/s00253-020-10567-2 32291487

[B4] Eagon R. G. (1962). *Pseudomonas natriegens*, a Marine Bacterium with a Generation Time of Less than 10 Minutes. J. Bacteriol. 83, 736–737. 10.1128/jb.83.4.736-737.1962 13888946 PMC279347

[B5] Ellis G. A. Tschirhart T. Spangler J. Walper S. A. Medintz I. L. Vora G. J. (2019). Exploiting the Feedstock Flexibility of the Emergent Synthetic Biology Chassis *Vibrio natriegens* for Engineered Natural Product Production. Mar. Drugs 17, 679. 10.3390/md17120679 PMC6950413 31801279

[B6] Erian A. M. Freitag P. Gibisch M. Pflügl S. (2020). High Rate 2,3-Butanediol Production with *Vibrio natriegens* . Bioresour. Technol. Rep. 10, 100408. 10.1016/j.biteb.2020.100408

[B7] Ge Y. Li K. Li L. Gao C. Zhang L. Ma C. (2016). Contracted but Effective: Production of Enantiopure 2,3-Butanediol by Thermophilic and GRAS *Bacillus licheniformis* . Green Chem. 18, 4693–4703. 10.1039/c6gc01023g

[B8] Guo Z.-W. Ou X.-Y. Liang S. Gao H.-F. Zhang L.-Y. Zong M.-H. (2020a). Recruiting a Phosphite Dehydrogenase/formamidase-Driven Antimicrobial Contamination System in *Bacillus subtilis* for Nonsterilized Fermentation of Acetoin. ACS Synth. Biol. 9, 2537–2545. 10.1021/acssynbio.0c00312 32786356

[B9] Guo Z.-W. Ou X.-Y. Xu P. Gao H.-F. Zhang L.-Y. Zong M.-H. (2020b). Energy- and Cost-Effective Non-Sterilized Fermentation of 2,3-Butanediol by an Engineered *Klebsiella pneumoniae* OU7 with an Anti-Microbial Contamination System. Green Chem. 22, 8584–8593. 10.1039/d0gc03044a

[B10] Hoff J. Daniel B. Stukenberg D. Thuronyi B. W. Waldminghaus T. Fritz G. (2020). *Vibrio natriegens* : an Ultrafast-Growing Marine Bacterium as Emerging Synthetic Biology Chassis. Environ. Microbiol. 22, 4394–4408. 10.1111/1462-2920.15128 32537803

[B11] Hoffart E. Grenz S. Lange J. Nitschel R. Müller F. Schwentner A. (2017). High Substrate Uptake Rates Empower *Vibrio natriegens* as Production Host for Industrial Biotechnology. Appl. Environ. Microbiol. 83, e01614–17. 10.1128/AEM.01614-17 28887417 PMC5666143

[B12] Levi P. G. Cullen J. M. (2018). Mapping Global Flows of Chemicals: from Fossil Fuel Feedstocks to Chemical Products. Environ. Sci. Technol. 52, 1725–1734. 10.1021/acs.est.7b04573 29363951

[B13] Li T. Chen X.-B. Chen J.-C. Wu Q. Chen G.-Q. (2014). Open and Continuous Fermentation: Products, Conditions and Bioprocess Economy. Biotechnol. J. 9, 1503–1511. 10.1002/biot.201400084 25476917

[B14] Meng W. Zhang L. Cao M. Zhang Y. Zhang Y. Li P. (2021). 2,3-Butanediol Synthesis from Glucose Supplies NADH for Elimination of Toxic Acetate Produced during Overflow Metabolism. Cell Discov. 7, 43. 10.1038/s41421-021-00273-2 34103474 PMC8187413

[B15] Meng W. Zhang Y. Cao M. Zhang W. Lü C. Yang C. (2020). Efficient 2,3-Butanediol Production from Whey Powder Using Metabolically Engineered *Klebsiella oxytoca* . Microb. Cell Fact. 19, 162. 10.1186/s12934-020-01420-2 32778112 PMC7419187

[B16] Peng Y. Han X. Xu P. Tao F. (2020). Next‐Generation Microbial Workhorses: Comparative Genomic Analysis of Fast-Growing *Vibrio* Strains Reveals Their Biotechnological Potential. Biotechnol. J. 15, 1900499. 10.1002/biot.201900499 32034937

[B17] Ramamurthy P. C. Singh S. Kapoor D. Parihar P. Samuel J. Prasad R. (2021). Microbial Biotechnological Approaches: Renewable Bioprocessing for the Future Energy Systems. Microb. Cell Fact. 20, 55. 10.1186/s12934-021-01547-w 33653344 PMC7923469

[B18] Sambrook J. Russell D. W. (2001). Molecular Cloning: A Laboratory Manual. New York: Cold Spring Harbor Laboratory.

[B19] Thoma F. Blombach B. (2021). Metabolic Engineering of *Vibrio natriegens* . Essays Biochem. 65, 381–392. 10.1042/EBC20200135 33835156 PMC8314017

[B20] Thomas V. C. Sadykov M. R. Chaudhari S. S. Jones J. Endres J. L. Widhelm T. J. (2014). A Central Role for Carbon-Overflow Pathways in the Modulation of Bacterial Cell Death. PLoS Pathog. 10, e1004205. 10.1371/journal.ppat.1004205 24945831 PMC4063974

[B21] Tschirhart T. Shukla V. Kelly E. E. Schultzhaus Z. NewRingeisen E. Erickson J. S. (2019). Synthetic Biology Tools for the Fast-Growing Marine Bacterium *Vibrio natriegens* . ACS Synth. Biol. 8, 2069–2079. 10.1021/acssynbio.9b00176 31419124

[B22] Wang Z. Tschirhart T. Schultzhaus Z. Kelly E. E. Chen A. Oh E. (2020). Melanin Produced by the Fast-Growing Marine Bacterium *Vibrio natriegens* through Heterologous Biosynthesis: Characterization and Application. Appl. Environ. Microbiol. 86, e02749–19. 10.1128/AEM.02749-19 31836580 PMC7028964

[B23] Weinstock M. T. Hesek E. D. Wilson C. M. Gibson D. G. (2016). *Vibrio natriegens* as a Fast-Growing Host for Molecular Biology. Nat. Methods 13, 849–851. 10.1038/nmeth.3970 27571549

[B24] Xin B. Tao F. Wang Y. Liu H. Ma C. Xu P. (2017). Coordination of Metabolic Pathways: Enhanced Carbon Conservation in 1,3-propanediol Production by Coupling with Optically Pure Lactate Biosynthesis. Metab. Eng. 41, 102–114. 10.1016/j.ymben.2017.03.009 28396036

[B25] Xu Y. Chu H. Gao C. Tao F. Zhou Z. Li K. (2014). Systematic Metabolic Engineering of *Escherichia coli* for High-Yield Production of Fuel Bio-Chemical 2,3-Butanediol. Metab. Eng. 23, 22–33. 10.1016/j.ymben.2014.02.004 24525331

[B26] Yu L. P. Wu F. Q. Chen G. Q. (2019). Next‐Generation Industrial Biotechnology‐Transforming the Current Industrial Biotechnology into Competitive Processes. Biotechnol. J. 14, 1800437. 10.1002/biot.201800437 30927495

[B27] Yue H. Ling C. Yang T. Chen X. Chen Y. Deng H. (2014). A Seawater-Based Open and Continuous Process for Polyhydroxyalkanoates Production by Recombinant *Halomonas campaniensis* LS21 Grown in Mixed Substrates. Biotechnol. Biofuels 7, 108. 10.1186/1754-6834-7-108

[B28] Zhang Y. Li Z. Liu Y. Cen X. Liu D. Chen Z. (2021). Systems Metabolic Engineering of *Vibrio natriegens* for the Production of 1,3-propanediol. Metab. Eng. 65, 52–65. 10.1016/j.ymben.2021.03.008 33722653

