# Supplementary material for: Non-Sterilized Fermentation of 2,3-Butanediol with Seawater by Metabolic Engineered Fast-Growing Vibrio natriegens
Source: Front Bioeng Biotechnol. 2022 Jul 12;10:955097. doi: 10.3389/fbioe.2022.955097 (PMC9315368; doi:10.3389/fbioe.2022.955097)
Supplement: Supplementary file 1 [file DataSheet1.docx]

***Supplementary Material***

**Non-sterilized fermentation of 2,3-butanediol with seawater by metabolic engineered fast-growing *Vibrio natriegens***

# Supplementary Figures and Tables

## 1. Supplementary Figures


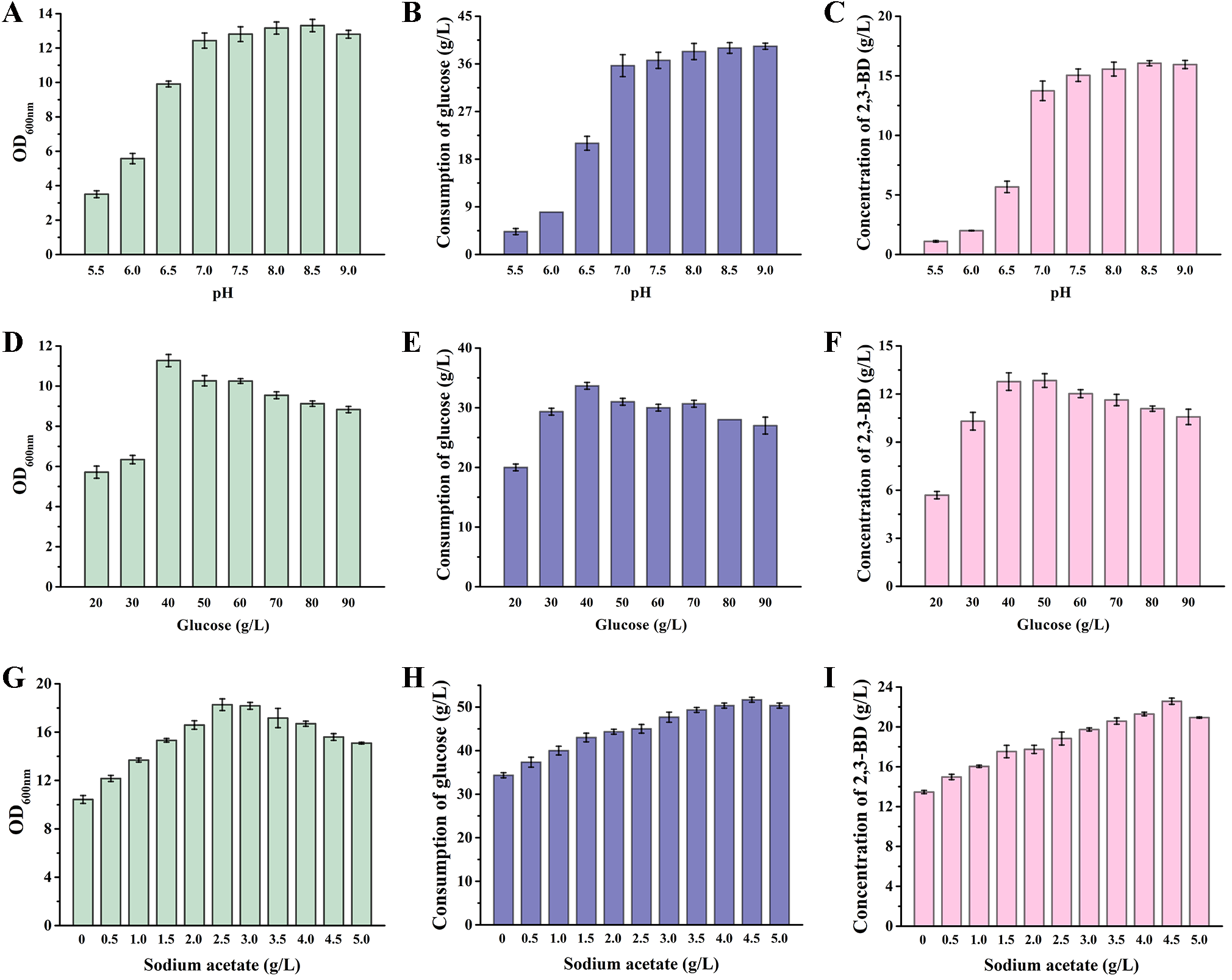


**Supplementary** **Figure S1.** Optimization of pH, initial glucose concentration, and addition of sodium acetate for 2,3-BD production by *V. natriegens*-pETRABC. Biomass **(A)**, consumption of glucose **(B)**, and concentration of 2,3-BD **(C)** in pH optimization. Biomass **(D)**, consumption of glucose **(E)**, and concentration of 2,3-BD **(F)** in initial glucose concentration optimization. Biomass **(G)**, consumption of glucose **(H)**, and concentration of 2,3-BD (**I**) in sodium acetate concentration optimization. The experiments were conducted in 300-mL flasks containing 50 mL of medium. Data shown are mean ± s.d. (n = 3 independent experiments).


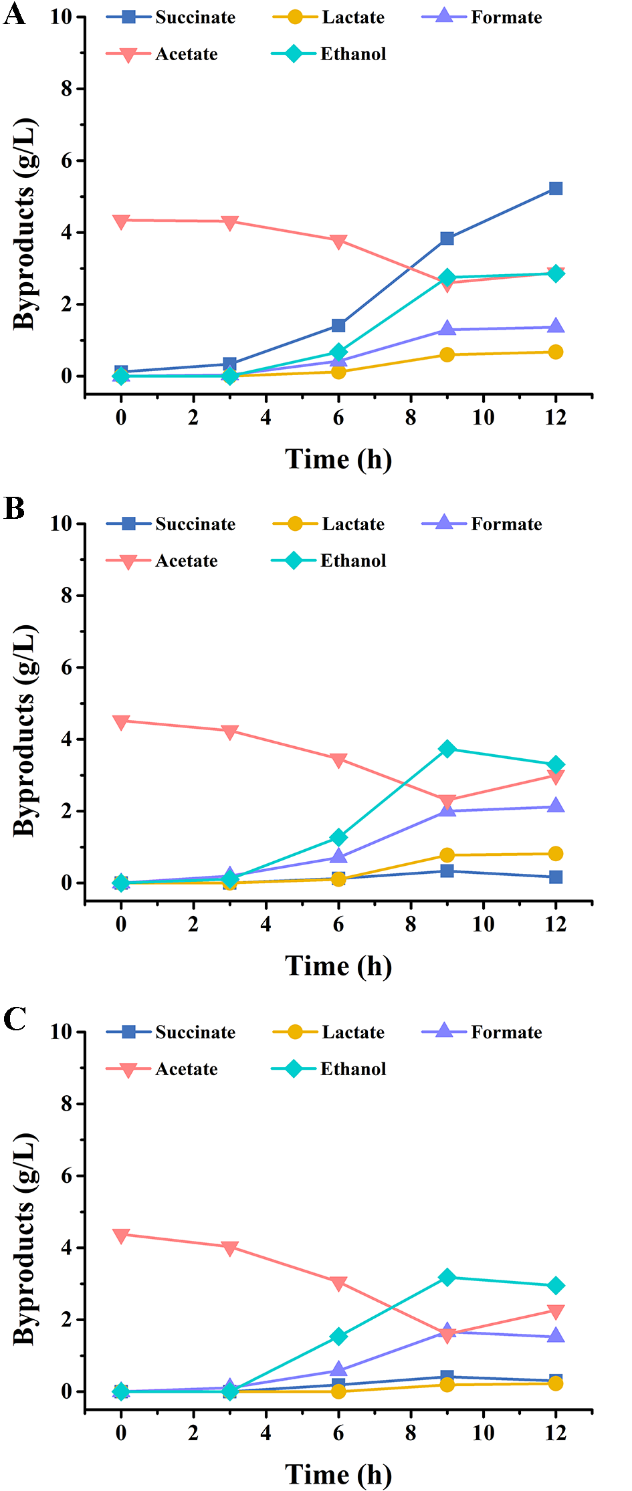


**Supplementary Figure S2.** Byproducts generation during non-sterilized batch fermentation of strain *V. natriegens*-pETRABC **(A)**, *V. natriegens*Δ*frdA*-pETRABC **(B)**, and *V. natriegens*Δ*frdA*Δ*ldhA*-pETRABC **(C)** in 1-L fermenter. The experiments were conducted in a 1-L fermenter containing 0.8 L of medium with an initial glucose concentration of 50 g/L.


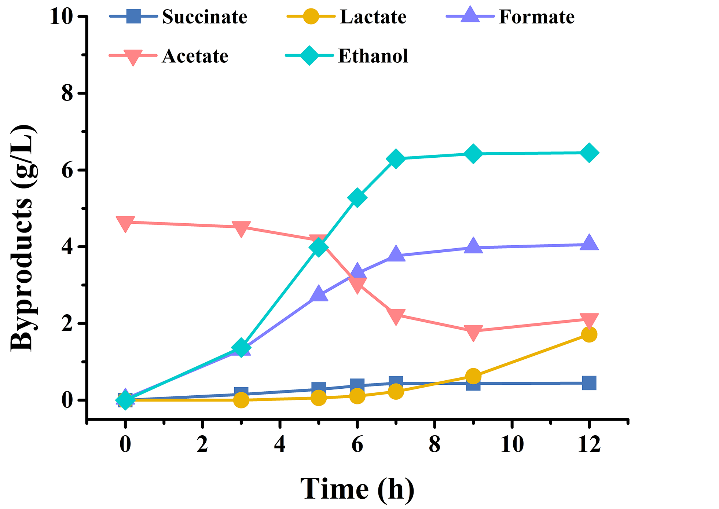


**Supplementary Figure S3.** Byproducts generation during non-sterilized fed-batch fermentation of strain *V. natriegens*Δ*frdA*Δ*ldhA*-pETRABC in 7.5-L fermenter. The experiments were conducted in a 7.5-L fermenter containing 5 L of medium with an initial glucose concentration of 50 g/L.

## 2. Supplementary Tables

**Supplementary** **Table S1.** Strains and plasmids used in this study.

| **Strain or plasmid** | **Relevant characteristics^a^** | **Origin** |
| --- | --- | --- |
| **Strain** |  |  |
| *Escherichia coli* S17-1 | *recA*, *pro*, *thi*, conjugative strain able to host λ-pir-dependent plasmids | Simon et al., 1983 |
| *E. coli* DH5α | F^–^ φ80*lacZ*∆M15 ∆(*lacZYA-argF*)U169 *deoR recA*1 *endA*1 *hsdR*17(r_K_^–^, m_K_^+^) *phoA* *supE*44 λ^–^ *thi-*1 *gyrA*96 *relA*1, used for gene clone | Novagen |
| *Vibrio natriegens* ATCC 14048 | Wild-type | ATCC |
| *E. coli* BL21-pETRABC | *E.* *coli* BL21 harboring pETRABC | Xu et al., 2014 |
| *V. natriegens*-pETRABC | *V. natriegens* ATCC 14048 harboring pETRABC | This study |
| *V. natriegens*-pET28a | *V. natriegens* ATCC 14048 harboring pET28a | This study |
| *V. natriegens*Δ*frdA* | *V.* *natriegens* ATCC 14048 with deletion of *frdA* | This study |
| *V. natriegens*Δ*frdA*Δ*ldhA* | *V.* *natriegens* ATCC 14048 with deletion of *frdA* and *ldhA* | This study |
| *V. natriegens*Δ*frdA*-pETRABC | *V. natriegens* ATCC 14048 with deletion of *frdA*, harboring pETRABC | This study |
| *V. natriegens*Δ*frdA*Δ*ldhA*-pETRABC | *V. natriegens* ATCC 14048 with deletion of *frdA* and *ldhA*, harboring pETRABC | This study |
| **Plasmid** |  |  |
| pET28a | Vector for gene expression; Km^r^ | Lab stock |
| pETRABC | Plasmid pET28a carrying 2,3-BD gene cluster with its operon and regulate protein *budR* originated from *E. cloacae* SDM, Km^r^ | Xu et al., 2014 |
| pKR6K_Cm_ | Cm^r^, gene replacement vector derived from plasmid pK18*mobsacB*, R6K origin, Mob^+^ *sac*B, and the Km^r^ resistance was replaced by Cm^r^ | Xin et al., 2017 |
| pKDΔ*frdA* | pKR6K_Cm_ derivative, carries partial lengths of *frdA* in *V. natriegens* ATCC 14048 | This study |
| pKDΔ*ldhA* | pKR6K_Cm_ derivative, carries partial lengths of *ldhA* in *V. natriegens* ATCC 14048 | This study |

^a^Km^r^, kanamycin resistance; Cm^r^, chloromycetin resistance.

**Supplementary Table S2.** The primers used in this study.

| Primer^a^ | Sequence (5′-3′)^b^ |
| --- | --- |
| PΔ*frdA*.f1 (EcoRI) | CCGGAATTCGTGCAAATTATCACCACAGA |
| PΔ*frdA*.r2 (overlap) | GCATCAGATCTTCTTGGCGGTAGGGTGTGAAGCATATGGA |
| PΔ*frdA*.f3 (overlap) | TCCATATGCTTCACACCCTACCGCCAAGAAGATCTGATGC |
| PΔ*frdA*.r4 (BamHI) | CGCGGATCCTTATGCTTGCTCCTCTGCGTTCTT |
| PΔ*ldhA*.f1 (EcoRI) | CCGGAATTCTAGGGTGAACGATCACAAGT |
| PΔ*ldhA*.r2 (overlap) | CCACGAAGACGGCGAAAATGTAACGTGCTGTTCACGGGT |
| PΔ*ldhA*.f3 (overlap) | ACCCGTGAACAGCACGTTACATTTTCGCCGTCTTCGTGG |
| PΔ*ldhA*.r4 (BamHI) | CGCGGATCCCTTCTTCTTGTTTATGACCT |

^a^“f” indicates that this is a sense primer; “r” indicates that this is an antisense primer.

^b^Restriction sites are underlined.

**References**

Xin, B., Tao, F., Wang, Y., Liu, H., Ma, C., and Xu, P. (2017). Coordination of metabolic pathways: enhanced carbon conservation in 1,3-propanediol production by coupling with optically pure lactate biosynthesis. *Metab. Eng.* 41, 102–114. doi: 10.1016/j.ymben.2017.03.009

Simon, R., Priefer, U., and Pühler, A. (1983). A broad host range mobilization system for in vivo genetic engineering: transposon mutagenesis in gram negative bacteria. *Nat. Biotechnol.* 1, 784–791. doi: 10.1038/nbt1183-784

Xu, Y., Chu, H., Gao, C., Tao, F., Zhou, Z., Li, K., et al. (2014). Systematic metabolic engineering of *Escherichia coli* for high-yield production of fuel bio-chemical 2,3-butanediol. *Metab. Eng.* 23, 22–33. doi: 10.1016/j.ymben.2014.02.004
